# Association Analysis of Bitter Receptor Genes in Five Isolated Populations Identifies a Significant Correlation between *TAS2R43* Variants and Coffee Liking

**DOI:** 10.1371/journal.pone.0092065

**Published:** 2014-03-19

**Authors:** Nicola Pirastu, Maarten Kooyman, Michela Traglia, Antonietta Robino, Sara M. Willems, Giorgio Pistis, Pio d’Adamo, Najaf Amin, Angela d’Eustacchio, Luciano Navarini, Cinzia Sala, Lennart C. Karssen, Cornelia van Duijn, Daniela Toniolo, Paolo Gasparini

**Affiliations:** 1 Institute for Maternal and Child Health, Istituto Di Ricovero e Cura a Carattere Scientifico “Burlo Garofolo,” Trieste, Italy; 2 Department of Medical, Surgical and Health Sciences, University of Trieste, Trieste, Italy; 3 Genetic Epidemiology Unit, Department of Epidemiology, Erasmus Medical Center, Rotterdam, The Netherlands; 4 illycaffè s.p.a, Trieste, Italy; 5 Centre for Medical Systems Biology, Leiden University Medical Center, Leiden, The Netherlands; 6 Division of Genetics and Cell Biology, San Raffaele Scientific Institute, Milano, Italy; German Institute for Human Nutrition, Germany

## Abstract

Coffee, one of the most popular beverages in the world, contains many different physiologically active compounds with a potential impact on people’s health. Despite the recent attention given to the genetic basis of its consumption, very little has been done in understanding genes influencing coffee preference among different individuals. Given its markedly bitter taste, we decided to verify if bitter receptor genes (*TAS2Rs*) variants affect coffee liking. In this light, 4066 people from different parts of Europe and Central Asia filled in a field questionnaire on coffee liking. They have been consequently recruited and included in the study. Eighty-eight SNPs covering the 25 *TAS2R* genes were selected from the available imputed ones and used to run association analysis for coffee liking. A significant association was detected with three SNP: one synonymous and two functional variants (W35S and H212R) on the TAS2R43 gene. Both variants have been shown to greatly reduce *in vitro* protein activity. Surprisingly the wild type allele, which corresponds to the functional form of the protein, is associated to higher liking of coffee. Since the hTAS2R43 receptor is sensible to caffeine, we verified if the detected variants produced differences in caffeine bitter perception on a subsample of people coming from the FVG cohort. We found a significant association between differences in caffeine perception and the H212R variant but not with the W35S, which suggests that the effect of the TAS2R43 gene on coffee liking is mediated by caffeine and in particular by the H212R variant.

No other significant association was found with other TAS2R genes. In conclusion, the present study opens new perspectives in the understanding of coffee liking. Further studies are needed to clarify the role of the TAS2R43 gene in coffee hedonics and to identify which other genes and pathways are involved in its genetics.

## Introduction

Coffee is one of the most widely drunk beverages in the world. It is second only to water and tea[1]. Given its widespread use and its content of different physiologically active compounds such as caffeine, polyphenols (eg chlorogenic acids), niacin, N-methylpyridinium ion and others [2], coffee has been studied in particular to verify its effects on health and to find possible relations with common diseases. In this light, it has been shown that coffee consumption has protective effects on various common pathologies such as cardiovascular diseases [3], hypertension [4], [5], Alzheimer’s and Parkinson’s diseases [6], [7], type 2 diabetes [8]–[10], some types of cancer [11], [12] and hearing functions [13], while it may predispose to sleep disturbances [14], [15]. Studies on the genetic bases of coffee consumption are quite old, and the first description of its heritability in Italy dates back to the 1960’s [16]. Recently, different independent genome-wide association studies carried out in Northern European populations have linked coffee and caffeine consumption to variations of different genes: CYP1A1-CYP1A2 [17], [18], AHR [17] NRCAM and ULK3 [18] while moderate association has been seen with the adenosine receptor A2, which is actually one of the effector proteins of caffeine [17].

Despite the recent observation that food hedonics might be a better predictor of long term food consumption rather than food frequency questionnaires[19], [20], very little has been done to understand which genetic factors influence coffee liking.

Coffee has a distinctive bitter taste and the perceived bitterness has been linked to a particular haplotype which includes polymorphism on *TAS2R3*, *TAS2R4* and *TAS2R5* [21], however this association did not have an impact on its liking. Further studies have tried to link coffee liking to phenylthiocarbamide (PTC) perception and *TAS2R38* genotypes. Although coffee bitterness shows positive correlation with PTC and PROP perception, no association was found with the *TAS2R38* gene [21]–[23]. Moreover, a recent genome wide association study has linked differences in caffeine detection thresholds to the TAS2R gene cluster on chromosome 12, although it failed to identify a functional variant explaining this difference[24]. Two large twins studies [25], [26] have shown that coffee liking had a strong genetic component (42% vs 62%), while most of the remaining variance was explained by unique environmental factors (respectively 58% and 38%). In contrast, the genetic component of coffee consumption is lower (respectively 42% and 39%) and also shared environmental components explain part of the variance. From a genetic point of view these results suggest that studying the hedonic aspect of coffee may produce better results compared to studying its consumption. Therefore, we decided to focus on the relationship between bitter taste perception genes and coffee liking: in particular to verify if any bitter taste receptor variant is associated with differences in coffee preference.

## Materials and Methods

### Study populations

Samples have been collected in various populations from Europe and Central Asia. More specifically our study includes: 402 individuals come from INGI-CARL a population coming from Carlantino, a small village located in Puglia (Southern Italy); 749 are defined as INGI-FVG, making reference to 6 villages situated in the Friuli Venezia Region in North-Eastern Italy and finally 1160 come from INGI-VB, i.e. a population coming from the Val Borbera Valley in North-Western Italy. The Erasmus Rucphen Family (ERF) study is a cross-sectional cohort including 3,000 living descendants of 22 couples who had at least 6 children baptized in the community church around 1850-1900; 1310 samples were used from this study. Finally, Silk Road (SR) is a cohort of ∼1000 individuals resulting from the sampling of 20 communities coming from 5 nations (Armenia, Azerbaijan, Georgia, Uzbekistan, Tajikistan and Kazakhstan) located along the Silk Road.

### Coffee liking ascertainment

Coffee liking was ascertained by asking each participant to rate coffee-liking on a 9-point scale in which 1 equals to “dislike extremely” whereas 9 equals to “like extremely”[27]. In order to assess individual liking in the SR population a 5-point scale coupled with smiley faces was used. This scale is commonly used in case of linguistic barriers or when working with illiterate people as was the case of the SR population [28]. Given the differences in the two scales, data have been standardized by dividing each score for the number of categories of the used scale, therefore 9 for the European populations and 5 for the SR study. Table S1 reports the demographic summary and the trait description for each population.

### Genotyping and imputation

Genotyping was carried out as previously described [29]–[31]. Briefly, INGI-CARL, INGI-FVG and INGI-VB have been genotyped with Illumina 370k high density SNP array, while SR has been genotyped with Illumina 700k high density SNP arrays. Genotype imputation on the INGI cohorts and SR was conducted after standard QC using SHAPEIT2 [32] for the phasing step and IMPUTE2 [33] for the imputation using the1000 Genomes phase I v3 reference set [34]. ERF has been genotyped with different genotyping platform: Illumina 318k, 350k, 610k and Affymetrix 200k. Genotypes were pooled together after QC, phased and imputed to the 1000Genomes dataset phase I v3 [34] using MaCH and minimac [35]. After imputation we excluded from the statistical analyses SNPs with MAF < 0.01 or Info < 0.4 for all populations but ERF for which R^2^<0.3 was used instead.

### Association analysis between coffee liking and selected SNPs

Association analysis was conducted using mixed model linear regression in which the standardized coffee liking was used as the dependent variable and the allele dosages as the independent variable. Sex and age were used as covariates. The kinship matrix based on all available genotyped SNPs was used as the random effect. For ERF the kinship matrix was estimated on 14.4k SNPs, common to all different genotyping platforms used. Association analysis was conducted using the GRAMMAR-Gamma method [36] as implemented in the GenABEL 1.7-2 [37]. R package was used to eliminate the effect of relatedness from the trait. MixABEL [37] was used for the actual association of the imputed SNPs. Only SNPs located inside the TAS2R genes which also passed post-imputation quality control were used for the association analysis. SNPs which did not pass quality control for more than one population were discarded as well. After these filtering steps, 89 SNPs were left for the statistical analyses. The number of SNPs used for each gene is reported in Table 1.

**Table 1:** Number of SNPs used for association testing in each gene.

Gene	N_SNPs_
*TAS2R1*	1
*TAS2R13*	2
*TAS2R14*	3
*TAS2R15*	8
*TAS2R16*	1
*TAS2R18*	7
*TAS2R19*	4
*TAS2R20*	11
*TAS2R3*	2
*TAS2R30*	6
*TAS2R31*	8
*TAS2R38*	3
*TAS2R4*	3
*TAS2R40*	1
*TAS2R41*	2
*TAS2R42*	8
*TAS2R43*	6
*TAS2R46*	3
*TAS2R5*	2
*TAS2R50*	3
*TAS2R60*	1
*TAS2R7*	1
*TAS2R8*	1
*TAS2R9*	1
*Total*	88

Association analysis was conducted separately for each population and results have been pooled together using the inverse-variance weighting method. In order to verify if the standardization of the traits was actually able to report them on the same scale, we performed metanalysis using the Stouffer z-score method which is based just on p-values and sample numerosity. We expected that if differences between the scale of the measure exist between the different populations, the two methods should give different results. No difference was detected in p-values between the two methods therefore we decided to report only the first, which has the advantage of giving also the effect- size estimates.

Since the size of the TAS2R genes is quite small, many SNPs are in strong Linkage Disequilibrium (LD). For this reason we decided to estimate the real number of tests performed in order to establish the significance threshold for our results. We calculated non-trivial eigenSNPs (nte) for each gene using the GRASS method [38] and used the sum of all the ntes as the number of total independent tests. This type of analysis revealed that we were performing only 40 independent tests and when applying Bonferroni correction significance, threshold was thus set at 1.25×10^−3^.

### Association between significant SNPs and caffeine bitterness perception

Individual caffeine bitterness perception was assessed in 151 subjects coming from the FVG cohort. Each participant was asked to rate the bitterness of a solution of 1 g/l caffeine, and of a commercially available canned coffee (Illy issimo Caffè No Sugar by Ilko Coffee International), using the LMS (labeled magnitude scale) [39]. The LMS is a quasi-logarithmic 100-mm scale anchored to the labels ‘barely taste it’, ‘weak’, ’moderate’, ‘strong’, ‘very strong’ or ‘strongest imaginable’. Participants were instructed first to the verbal descriptors of the scale and also to make a mark anywhere on the scale, not only near the descriptors.

In order to avoid biases in the coffee tasting, the coffee solution was presented unbranded. To assess general taste sensitivity, we asked subjects to rate also a solution 0.1 M of NaCl on the same LMS scale.

Association analysis was conducted using a mixed model regression as implemented in the polygenic function of GenABEL. Since taste sensitivity was measured for all solutions on a logaritmic scale, as far as regression is concerned, they were all transformed using the log10 of the measure. The final regression model included sex, age, NaCl perception and coffee bitterness perception. This last variable was included in order to distinguish specific caffeine bitterness perception from general coffee bitter perception.

Given the limited number of samples available, we tested association only between the SNPs which resulted significantly associated to coffee liking.

### Ethical statement

All studies adhered to the tenets of the Declaration of Helsinki. The ERF study was approved by the Medical Ethics Committee of the Erasmus Medical Center in Rotterdam. Informed consent was obtained after explanation of the nature and possible consequences of the study.

All subjects in the INGI-CARL, INGI-FVG and SR studies provided written informed consent before participation. Approval for the research protocol was obtained from the ethical committee of IRCCS-Burlo Garofolo Hospital.

The VB study, including the overall plan and the informed consent form was reviewed and approved by the institutional review boards of San Raffaele Hospital in

Milan and by the ethical committee of the Regional Authorities of Piemonte.

## Results

Association results for all SNPs used in the analyses are shown in Table S2. After multiple testing correction, we found two SNPs significantly associated to coffee liking, rs71443637 (p = 4.9×10^−4^) and rs35720106 (p = 6.9×10^−4^) on the TAS2R43 gene.

rs68157013 also on the TAS2R43 gene was found to be close to significantly associated to coffee liking with a p of 1.7×10^−3^. No other significant association was found on any of the other tested genes. Given that these 3 SNPs had been excluded only on the ERF population because of the low Rsq, we decided to retrieve them and add them to the analyses.

**Figure 1:**
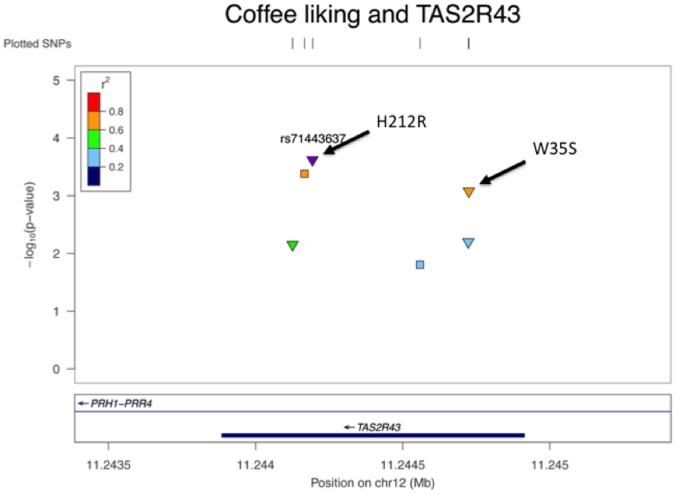
Regional plot of the association results in the *TAS2R43* gene. Triangles represent non-synonymous SNPs whereas squares represent synonymous SNPs.. The colors represent the LD between the SNPs and rs71443637 with respect to the 1000 Genomes CEU population. The violet point is the index SNP which the LD refers to. The plot was created with the LocusZoom software [40].

All SNPs showed and improvement in the association with a combined p of 9.2×10^−4^ for rs68157013, 2.7×10^−4^ for rs71443637 and 4.3×10^−4^ for rs35720106. The first two SNPs are non-synonymous variants leading to amino acid changes, W35S and H212R respectively. In particular the wild type alleles (C for rs68157013 and T for rs71443637) are associated to higher liking of coffee. Results for rs68157013 and rs71443637 are shown in Table 2. The SNP rs35720106 is a synonymous one, and its association is most likely due to the strong LD with rs71443637 (r^2^≈0.75 ). A regional plot of the results on TAS2R43 gene is shown in Figure 1 [40].

**Table 2:** Association results between coffee liking and the two non-synonymous SNPs.

	Population	beta	*p*
rs68157013 W35R	INGI-CARL	0.04	6.07×10^−3^
	INGI-FVG	0.01	6.27×10^−1^
	INGI-VB	0.02	8.66×10^−2^
	ERF	0.05	1.90×10^−1^
	SR	0.04	6.95×10^−2^
	pooled	0.02	9.17×10^−4^
rs71443637 H212R	INGI-CARL	0.04	3.18×10^−3^
	INGI-FVG	0.00	7.02×10^−1^
	INGI-VB	0.02	3.23×10^−2^
	ERF	0.04	2.43×10^−1^
	SR	0.04	5.79×10^−2^
	pooled	0.02	2.68×10^−4^

beta represents the coefficient of the linear regression with respect to the wild type allele W for rs68157013 and H for rs71443637 *p* is the p-value of the association analysis. All populations show concordant effect directions except for INGI-FVG which shows no apparent effect of the alleles on coffee liking.

**Figure 2:**
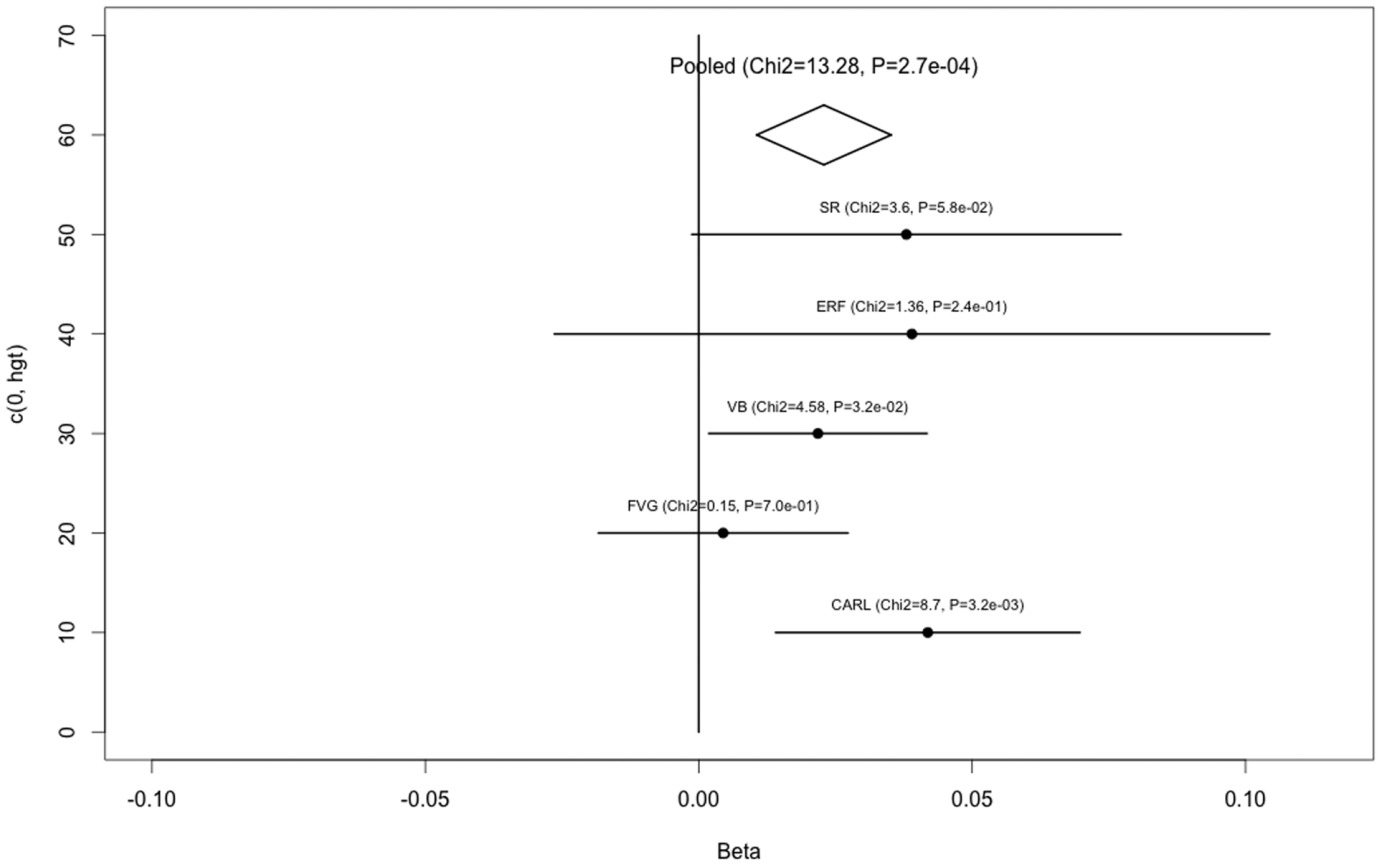
Forest plot of the normalized effect for the various populations for rs71443637. Points represent the effect estimates and bars indicate is the 95% confidence interval. The diamond represents the pooled effect. For each population *p*-values are also shown. INGI-FVG clearly shows no effect of the SNP on coffee liking.

Given that rs71443637 shows a stronger association as compared to the other ones, Figure 2 shows the forest plot for the different populations for this marker. Strikingly, the SNP has a very similar effect across the different populations except for INGI-FVG in which rs71443637 seems to have no effect. Overall rs71443637 explains 0.32% of the total variance.

Testing the three identified SNPs with perceived caffeine bitterness in the FVG sub-sample showed significant association with rs71443637 (p = 2.7×10^−3^) and rs68157013 (p = 0.01) while we detected no association with rs35720106 (p = 0.17). Similarly to what was found on coffee liking, T allele of rs71443637 was associated with a higher perception of caffeine bitterness, thus explaining an overall 5.7% of caffeine bitter perception.

## Discussion

In this work we describe a novel association of a functional variant of TAS2R43 with coffee liking and differences in caffeine bitter perception. The results on caffeine are consistent with a recent genome wide association study that has identified the bitter receptor gene cluster on chromosome 12 as being strongly associated to caffeine thresholds, explaining up to 8,9% of the variation in caffeine perceived bitterness [24]. The *TAS2R43* receptor could contribute to the bitter aftertaste of sulfonyl amide sweeteners bitter aftertaste [41] and, more importantly, together with *TAS2R7*, *TAS2R10*, *TAS2R14* and *TAS2R46*, it is activated by caffeine in *in vitro* studies [42]. The associated variants we found, produce two amino acid changes (W35S and H212R), which cause largely diminished protein functionality [43]. In particular, Pronin *et al.* compare three different proteins: *hTAS2R43-WH* which carries tryptophan and histidine at positions 35 and 212 respectively, *hTAS2R43-SH* which carries serine at position 35 and arginine at position 212 and finally *hTAS2R43-SR* with arginine in position 212. While *hTAS2R43-WH* shows good activity when stimulated with aloin, both *hTAS2R43-SH* and *hTAS2R43-SR* responded very weakly, thus suggesting that the amino acid substitution W35R is the factor leading to the observed difference. In our study we found that H212R variant shows a stronger association than W35R on coffee liking while we detected no association between the W35R variant and caffeine perceived bitterness. Many possible explanations may account for this difference. While W35R is important for aloin recognition H212R could be responsible for the activation by caffeine, however further functional studies are needed to unravel this issue.

Nevertheless, our results clearly demonstrate that people carrying the non-functional allele show lower liking for coffee and lower caffeine perception. Although the percentage of variance explained by the identified variant appears to be small (0.32% of coffee liking and 5.6% of caffeine perceived bitterness), this result is in line with previous outcomes. For example, although TAS2R38 explains up to 49% of PTC bitter perception [44], its effect on cruciferous vegetables consumption is extremely small (∼0.8%) [45]. The fact that people capable of perceiving caffeine bitterness through hTAS2R43 actually show increased preference for coffee may seem in contrast with the bitter-equal-aversion paradigm, however we must consider it in the light of flavor-nutrient learning. Although this effect in humans is still controversial [46], our results seem to suggest that this relationship actually exists. In this particular case it is possible that people associate coffee’s positive effects to the particular bitterness given by caffeine, thus explaining the apparent paradox between higher perception of bitterness and higher liking. Our results are consistent with the previously observed increase in liking of beverages due to caffeine [47], [48]. This is usually obtained with near- or below- threshold concentrations and it is thought to be due to some other interaction, for example with the adenosine receptor [49]. The present findings, however, may lead to a different interpretation, suggesting that this effect might be mediated by TAS2R43. Among compounds which are known to activate TAS2R43 [42] only caffeine is present in coffee, therefore, although we cannot exclude that other untested bitter compounds with physiological positive effects could be responsible for the observed association, our results suggests that, at least for TAS2R43, caffeine could actually be responsible for differences in liking.

The lack of associations between the other bitter receptors and coffee liking is consistent with previously described research [21]. Our findings not only describe the first association between a bitter receptor and coffee liking, but show also that this relationship is probably mediated by caffeine in the context of flavor-nutrient learning. Moreover, the study implies that we are able to distinguish one bitter compounds from another and that when we commonly refer to bitter taste, we are referring to a range of sensations mediated by a wide variety of molecules and receptors.

Roudnitzky et al. [50] have described a long range haplotype spanning most of the TAS2R gene cluster. Given the use of imputed SNPs we were unable to assess the presence of long-range haplotypes in our samples. However such a shared-across-populations’ haplotype is unlikely to exist since no further association signal was observed in the same locus. This also suggests that using such different populations was extremely important for fine mapping of the identified locus.

Finally the H212R variant explains little of the total variance of coffee liking (r2≈ 0.4%), further confirming that genome-wide studies (GWAS) on larger numbers of samples are needed to clarify which genes/receptors/pathways are determining people’s preferences for coffee.

In conclusion, the present study is a good starting point to understand which genes may be involved in coffee liking, thus opening new perspectives for food industries and nutritionists.

## Supporting Information

Table S1 **Cohort descriptives.**
(DOCX)

Table S2 **Complete association results.**
(DOCX)
