# Supplementary material for: Association Analysis of Bitter Receptor Genes in Five Isolated Populations Identifies a Significant Correlation between TAS2R43 Variants and Coffee Liking
Source: PLoS One. 2014 Mar 19;9(3):e92065. doi: 10.1371/journal.pone.0092065 (PMC3960174; doi:10.1371/journal.pone.0092065)
Supplement: Table S1 — Cohort descriptives. (DOCX) [file pone.0092065.s001.docx]

| **Population** | **Number of samples** | **Mean Age (SD)** | **Percentage of women** | **Mean Coffee liking (SD)** |
| --- | --- | --- | --- | --- |
| INGI-CARL | 402 | 52.56(17.26) | 58% | 0.83(0.18) |
| INGI-FVG | 749 | 50.85(15.80) | 59% | 0.81(0.18) |
| INGI-VB | 1160 | 53.23(16.62) | 63% | 0.82(0.21) |
| ERF | 1310 | 47.4 (13.23) | 55% | 0.75(0.21) |
| SR | 445 | 39.12(15.86) | 59% | 0.76(0.27) |

Table S1. Cohort descriptive. The first column indicates the cohort name, the second the number of samples used for the analyses, the third column represents mean age and standard deviation, the fourth the percentage of women in each sample, and the fifth the mean standardized coffee liking and standard deviation.
