# Supplementary material for: Association Analysis of Bitter Receptor Genes in Five Isolated Populations Identifies a Significant Correlation between TAS2R43 Variants and Coffee Liking
Source: PLoS One. 2014 Mar 19;9(3):e92065. doi: 10.1371/journal.pone.0092065 (PMC3960174; doi:10.1371/journal.pone.0092065)
Supplement: Table S2 — Complete association results. (DOCX) [file pone.0092065.s002.docx]

| SNP | **CHR** | **Position** | **OA** | **EA** | ***N*** | **beta** | **sebeta** | **Direction** | ***p*** | **Gene** |
| --- | --- | --- | --- | --- | --- | --- | --- | --- | --- | --- |
| rs2234233 | 5 | 9629529 | G | A | 4065 | 7.0E-03 | 6.8E-03 | +-+++ | 3.07E-01 | TAS2R1 |
| rs860170 | 7 | 122635024 | T | C | 4065 | 8.7E-03 | 4.9E-03 | +++++ | 7.37E-02 | TAS2R16 |
| rs765007 | 7 | 141463914 | T | C | 4066 | 6.7E-04 | 4.6E-03 | +-++- | 8.83E-01 | TAS2R3 |
| rs2270009 | 7 | 141464765 | C | T | 4066 | 2.4E-04 | 4.5E-03 | +-++- | 9.58E-01 | TAS2R3 |
| rs2233998 | 7 | 141478308 | T | C | 4066 | 1.2E-03 | 4.5E-03 | +-++- | 7.90E-01 | TAS2R4 |
| rs2234001 | 7 | 141478574 | G | C | 4066 | 1.7E-03 | 4.5E-03 | +-++- | 7.15E-01 | TAS2R4 |
| rs2234002 | 7 | 141478800 | A | G | 4066 | -1.5E-03 | 4.5E-03 | -+--+ | 7.44E-01 | TAS2R4 |
| rs2234012 | 7 | 141490107 | A | G | 4066 | 1.0E-03 | 4.6E-03 | --++- | 8.26E-01 | TAS2R5 |
| rs2227264 | 7 | 141490238 | G | T | 4065 | 1.5E-03 | 4.6E-03 | --++- | 7.52E-01 | TAS2R5 |
| rs10246939 | 7 | 141672604 | T | C | 4063 | -2.3E-03 | 4.6E-03 | +-+-+ | 6.12E-01 | TAS2R38 |
| rs1726866 | 7 | 141672705 | A | G | 4065 | -2.3E-03 | 4.5E-03 | +++-- | 6.06E-01 | TAS2R38 |
| rs713598 | 7 | 141673345 | C | G | 4066 | -1.6E-03 | 4.7E-03 | +-+-- | 7.33E-01 | TAS2R38 |
| rs10260248 | 7 | 142919731 | C | A | 2756 | -1.1E-02 | 9.6E-03 | +?--+ | 2.59E-01 | TAS2R40 |
| rs4595035 | 7 | 143141475 | C | T | 4065 | 4.6E-03 | 5.1E-03 | ++++- | 3.71E-01 | TAS2R60 |
| rs1404635 | 7 | 143175154 | G | A | 4065 | 1.6E-03 | 5.2E-03 | -++-- | 7.58E-01 | TAS2R41 |
| rs10278721 | 7 | 143175345 | C | T | 4065 | 1.7E-03 | 5.2E-03 | -++-- | 7.47E-01 | TAS2R41 |
| rs619381 | 12 | 10954258 | C | T | 2753 | 2.8E-03 | 9.0E-03 | +?-++ | 7.57E-01 | TAS2R7 |
| rs1548803 | 12 | 10959031 | C | T | 4065 | -4.6E-03 | 5.2E-03 | --+-- | 3.82E-01 | TAS2R8 |
| rs3741845 | 12 | 10962115 | G | A | 4059 | 5.8E-03 | 5.0E-03 | ++-++ | 2.45E-01 | TAS2R9 |
| rs1015442 | 12 | 11060695 | T | C | 4062 | -6.5E-03 | 4.7E-03 | --+-- | 1.66E-01 | TAS2R13 |
| rs1015443 | 12 | 11061122 | C | T | 4063 | 6.3E-03 | 4.7E-03 | ++-++ | 1.79E-01 | TAS2R13 |
| rs3851584 | 12 | 11090077 | G | T | 4066 | -6.1E-03 | 5.2E-03 | --+-- | 2.41E-01 | TAS2R14 |
| rs3741843 | 12 | 11091432 | T | C | 4064 | 3.9E-03 | 7.0E-03 | -+-+- | 5.80E-01 | TAS2R14 |
| rs7138535 | 12 | 11091693 | T | A | 4066 | 4.4E-03 | 6.3E-03 | ++-++ | 4.82E-01 | TAS2R14 |
| rs11054092 | 12 | 11117159 | T | C | 2756 | 6.0E-03 | 6.6E-03 | +?-++ | 3.64E-01 | TAS2R15 |
| rs11054093 | 12 | 11117214 | G | C | 2756 | 6.0E-03 | 6.6E-03 | +?-++ | 3.64E-01 | TAS2R15 |
| rs11054094 | 12 | 11117292 | G | A | 2756 | 6.0E-03 | 6.6E-03 | +?-++ | 3.64E-01 | TAS2R15 |
| rs4763599 | 12 | 11117321 | A | G | 2756 | 6.0E-03 | 6.6E-03 | +?-++ | 3.64E-01 | TAS2R15 |
| rs17810798 | 12 | 11117432 | G | C | 2756 | 6.0E-03 | 6.6E-03 | +?-++ | 3.64E-01 | TAS2R15 |
| rs11054095 | 12 | 11117631 | G | C | 2756 | 6.0E-03 | 6.6E-03 | +?-++ | 3.64E-01 | TAS2R15 |
| rs11054096 | 12 | 11117796 | T | C | 2756 | 5.3E-03 | 6.7E-03 | +?-++ | 4.35E-01 | TAS2R15 |
| rs11054097 | 12 | 11117857 | G | C | 2756 | 6.0E-03 | 6.6E-03 | +?-++ | 3.64E-01 | TAS2R15 |
| rs10772397 | 12 | 11138683 | C | T | 4064 | -7.0E-03 | 5.2E-03 | --+-- | 1.81E-01 | TAS2R50 |
| rs1376251 | 12 | 11138852 | C | T | 4066 | -1.3E-02 | 5.0E-03 | --+-- | 1.25E-02 | TAS2R50 |
| rs66679979 | 12 | 11138935 | T | C | 2756 | 5.0E-03 | 7.8E-03 | -?-++ | 5.19E-01 | TAS2R50 |
| rs61912291 | 12 | 11149390 | T | G | 4066 | 7.0E-03 | 6.5E-03 | ++-++ | 2.83E-01 | TAS2R20 |
| rs1450839 | 12 | 11149532 | A | G | 4066 | -1.4E-02 | 5.0E-03 | --+-- | 6.65E-03 | TAS2R20 |
| rs10845279 | 12 | 11149711 | A | C | 4063 | 1.3E-02 | 4.9E-03 | ++-++ | 5.94E-03 | TAS2R20 |
| rs10845280 | 12 | 11149720 | A | G | 4066 | -1.3E-02 | 4.9E-03 | --+-- | 6.67E-03 | TAS2R20 |
| rs10845281 | 12 | 11149769 | T | C | 4066 | -1.3E-02 | 4.9E-03 | --+-- | 6.66E-03 | TAS2R20 |
| rs12226919 | 12 | 11150033 | T | G | 4065 | 1.3E-02 | 4.9E-03 | ++-++ | 5.86E-03 | TAS2R20 |
| rs12226920 | 12 | 11150046 | T | G | 4063 | 1.3E-02 | 4.9E-03 | ++-++ | 6.10E-03 | TAS2R20 |
| rs79420812 | 12 | 11150054 | C | T | 4066 | -1.7E-02 | 7.2E-03 | ----- | 2.11E-02 | TAS2R20 |
| rs11054142 | 12 | 11150214 | G | A | 4066 | -1.3E-02 | 4.9E-03 | --+-- | 6.49E-03 | TAS2R20 |
| rs7135018 | 12 | 11150240 | T | C | 4066 | 6.8E-03 | 6.3E-03 | ++-++ | 2.76E-01 | TAS2R20 |
| rs11054143 | 12 | 11150319 | T | C | 4066 | -1.3E-02 | 4.9E-03 | --+-- | 6.64E-03 | TAS2R20 |
| rs10772420 | 12 | 11174276 | G | A | 4066 | 8.7E-03 | 4.6E-03 | +-+++ | 5.63E-02 | TAS2R19 |
| chr12:11174327 | 12 | 11174327 | C | T | 2756 | 9.9E-03 | 6.8E-03 | +?-++ | 1.46E-01 | TAS2R19 |
| rs1868769 | 12 | 11174753 | G | A | 4066 | -8.1E-03 | 6.1E-03 | --+-+ | 1.79E-01 | TAS2R19 |
| rs12313469 | 12 | 11175087 | G | A | 4066 | 8.2E-03 | 4.6E-03 | +-+++ | 7.22E-02 | TAS2R19 |
| rs12318612 | 12 | 11183108 | G | C | 4066 | 5.3E-03 | 5.4E-03 | +-+++ | 3.25E-01 | TAS2R31 |
| rs116737741 | 12 | 11183191 | T | C | 4066 | 1.3E-02 | 7.6E-03 | -+-++ | 8.81E-02 | TAS2R31 |
| rs10772423 | 12 | 11183217 | C | T | 4066 | 8.5E-03 | 4.6E-03 | +--++ | 6.68E-02 | TAS2R31 |
| rs10845293 | 12 | 11183255 | G | A | 4066 | 8.3E-03 | 4.6E-03 | +-+++ | 7.06E-02 | TAS2R31 |
| rs10845294 | 12 | 11183286 | G | C | 4066 | 5.3E-03 | 5.4E-03 | +-+++ | 3.24E-01 | TAS2R31 |
| rs10743938 | 12 | 11183451 | A | T | 4066 | -1.1E-02 | 5.9E-03 | --+-+ | 6.97E-02 | TAS2R31 |
| rs12370363 | 12 | 11183512 | A | G | 4066 | 6.4E-03 | 5.6E-03 | ++-++ | 2.54E-01 | TAS2R31 |
| rs10845295 | 12 | 11183832 | G | A | 4066 | 8.0E-03 | 4.7E-03 | +--++ | 8.88E-02 | TAS2R31 |
| rs2708381 | 12 | 11214145 | C | T | 4066 | 6.6E-03 | 6.0E-03 | ++-++ | 2.71E-01 | TAS2R46 |
| rs2708380 | 12 | 11214212 | A | T | 4066 | 1.2E-02 | 5.4E-03 | +-+++ | 2.57E-02 | TAS2R46 |
| rs73260771 | 12 | 11214360 | C | T | 4066 | 7.2E-03 | 5.5E-03 | +-+++ | 1.91E-01 | TAS2R46 |
| rs113410031 | 12 | 11244126 | G | A | 2756 | 1.8E-02 | 6.1E-03 | +?+++ | 3.94E-03 | TAS2R43 |
| **rs35720106** | **12** | **11244166** | **G** | **C** | **4066** | **-2.3E-02** | **-2.3E-02** | **-----** | **4.30E-04** | **TAS2R43** |
| **rs71443637** | **12** | **11244194** | **T** | **C** | **4066** | **-2.3E-02** | **-2.3E-02** | **-----** | **2.70E-04** | **TAS2R43** |
| rs144614950 | 12 | 11244559 | C | T | 2756 | 1.8E-02 | 7.0E-03 | +?+++ | 1.09E-02 | TAS2R43 |
| rs191086711 | 12 | 11244723 | A | C | 2756 | 2.0E-02 | 6.8E-03 | +?+++ | 3.94E-03 | TAS2R43 |
| **rs68157013** | **12** | **11244725** | **C** | **G** | **4066** | **-2.1E-02** | **-2.1E-02** | **-----** | **9.20E-04** | **TAS2R43** |
| rs75056416 | 12 | 11285580 | A | C | 2007 | -5.1E-03 | 1.7E-02 | +??-+ | 7.59E-01 | TAS2R30 |
| rs7313796 | 12 | 11285689 | A | C | 4066 | 4.1E-03 | 5.7E-03 | ++-++ | 4.77E-01 | TAS2R30 |
| rs2600357 | 12 | 11285754 | G | A | 4066 | 9.8E-03 | 4.5E-03 | +-+++ | 3.17E-02 | TAS2R30 |
| rs2600356 | 12 | 11285802 | G | T | 4066 | 9.3E-03 | 4.6E-03 | +-+++ | 4.54E-02 | TAS2R30 |
| rs2599404 | 12 | 11286088 | A | C | 4066 | 9.8E-03 | 4.6E-03 | +-+++ | 3.16E-02 | TAS2R30 |
| rs2600355 | 12 | 11286790 | A | C | 4066 | 9.6E-03 | 4.8E-03 | +-+++ | 4.29E-02 | TAS2R30 |
| rs7310047 | 12 | 11311520 | G | A | 4066 | 9.8E-03 | 4.5E-03 | +-+++ | 3.13E-02 | TAS2R18 |
| rs7296270 | 12 | 11311590 | A | T | 4066 | -7.0E-03 | 6.7E-03 | --+-+ | 2.96E-01 | TAS2R18 |
| rs61928603 | 12 | 11311787 | C | T | 4066 | -1.0E-02 | 6.5E-03 | --+-+ | 1.12E-01 | TAS2R18 |
| 12:11311936:I | 12 | 11311936 | S | L | 4066 | -7.0E-03 | 6.7E-03 | --+-+ | 2.97E-01 | TAS2R18 |
| rs61928604 | 12 | 11311947 | C | T | 4066 | -7.0E-03 | 6.7E-03 | --+-+ | 2.96E-01 | TAS2R18 |
| rs2290318 | 12 | 11311958 | G | C | 4066 | -1.5E-02 | 4.9E-03 | --+-- | 2.11E-03 | TAS2R18 |
| rs2290319 | 12 | 11312026 | C | A | 4066 | -1.5E-02 | 4.9E-03 | --+-- | 2.11E-03 | TAS2R18 |
| rs1650017 | 12 | 11338613 | C | G | 4066 | -7.6E-03 | 6.4E-03 | --+-+ | 2.39E-01 | TAS2R42 |
| rs1669411 | 12 | 11338614 | A | G | 4066 | -7.0E-03 | 6.4E-03 | --+-+ | 2.79E-01 | TAS2R42 |
| rs1669412 | 12 | 11338669 | C | T | 4066 | 6.7E-03 | 5.3E-03 | ++-++ | 2.09E-01 | TAS2R42 |
| rs1451772 | 12 | 11338750 | T | C | 4065 | 6.7E-03 | 5.3E-03 | ++-++ | 2.08E-01 | TAS2R42 |
| rs1669413 | 12 | 11338781 | T | G | 4065 | 7.5E-03 | 6.5E-03 | ++-+- | 2.48E-01 | TAS2R42 |
| rs5020531 | 12 | 11338957 | A | G | 4066 | -9.1E-03 | 4.7E-03 | --+-- | 5.36E-02 | TAS2R42 |
| rs1650019 | 12 | 11338983 | T | C | 4066 | -7.3E-03 | 6.5E-03 | --+-+ | 2.61E-01 | TAS2R42 |
| rs35969491 | 12 | 11339020 | T | A | 4066 | -9.1E-03 | 4.7E-03 | --+-- | 5.37E-02 | TAS2R42 |

Table S2. Meta analysis results for all studied SNPs. The SNP column indicates the markers name, CHR : the chromosome, Position: the chromosomal position expressed in base pairs, EA :effect allele, OA: other allele, N :total number of the samples, p :p-value of the meta analysis, Direction: the direction of the beta for the each study population in the following order INGI-CARL, ERF ,INGI-FVG,INGI-SR and INGI-VB.A question mark indicates that that particular SNP was discarded in that specific population. Gene: the gene the SNP is in. The significant SNPs are indicated in bold
